# Beauty is in the eye of the employer: Labor market discrimination of accountants

**DOI:** 10.3389/fpsyg.2022.928451

**Published:** 2022-07-29

**Authors:** Offer Moshe Shapir, Zeev Shtudiner

**Affiliations:** ^1^Department of Business, New York University Shanghai, Shanghai, China; ^2^Department of Economics and Business Administration, Ariel University, Ariel, Israel

**Keywords:** beauty, discrimination, labor market, accounting, decision-making

## Abstract

This research investigates labor market discrimination based on physical appearance in Israel’s Certified Public Accountant firms. Using a survey questionnaire, we showed that accountants in managerial positions prefer to hire more physically attractive candidates. This beauty premium is larger among the five biggest Certified Public Accountant firms and can be explained by the perception that attractive candidates possess essential traits for becoming successful accountants. An important implication of our results is that even among accounting firms, where professionalism is well defined, discrimination against candidates based on traits such as physical appearance can ineffectively eliminate suitably qualified interns.

## Introduction

We investigated the role of beauty in the hiring process in the field of accounting. Job opportunities in the accounting profession are highly competitive. In Britain, the accounting sector had the highest average number of applications per employer in 2013–2014 among all sectors (High Fliers, 2014), and in Israel, only 50% of accounting graduates find internships in Certified Public Accountant (CPA) firms. Due to this tough competition, CPA firms, and in particular the biggest firms, can choose their interns from a large pool of candidates. This competitiveness can result in employers looking for stronger quality signals (such as academic institution, grades, fluency in speech, and clothing), and physical appearance can be perceived as such a signal (because the candidates would be required to deal with clients on a regular basis).

There is a large gap between the office activity of the five big firms (i.e., EY, KPMG, Deloitte, PWC, and BDO; hereafter, Big 5 firms) versus the other ranked firms. For illustration, the total number of employees in the five firms of the second quintile does not exceed the total number of employees employed by BDO, the 5th-ranked firm. The Big 5 firms are responsible for the audits of 87% of the public companies in Israel. Big-company positions are more competitive^1^ and managers may use signals such as a candidate’s physical appearance and the associations it evokes to determine whether the candidate is qualified for the job.

Two separate questionnaires were designed to test whether physical attractiveness was a deciding factor in hiring. As part of the first stage of the study, accounting students from several universities in Israel (i.e., Tel Aviv, Bar Ilan, Hebrew, Ariel) evaluated photographs of individuals for their attractiveness and the six most essential traits for accountants (Davis et al., 2010), namely, ethics, analytics, detail-orientation, criticism, inquisition, and intuition. As part of our second stage, accountants in managerial positions were shown the ranked candidate photographs, and they rated their interest in hiring each candidate.

We found that candidates who are more attractive are more likely to be accepted to accounting internships. Women and men alike enjoy this beauty premium, and in the Big 5 firms, it is significantly higher. Moreover, we found that attractive people are believed to possess the six desirable characteristics of successful accountants (to a greater extent, compared with unattractive people). The rest of the article is organized as follows. A literature review is presented and the questionnaire design is discussed. We presented the empirical evidence and discussed the results. Finally, concluding remarks are offered.

## Literature review

There is scholarly agreement that attractiveness affects decision-making in the labor market. The first research to show that attractive people earn more than average-looking people across occupations in the labor market comes from Hamermesh and Biddle (1994) and Mobius and Rosenblat (2006) designed a laboratory-based labor market experiment to show that attractive workers from both genders are offered higher salaries. However, they did not find any evidence that good-looking workers perform better. In their study on ultimatum-game (bargaining environment) decisions, Solnick and Schweitzer (1999) found that attractive and unattractive participants make similar decisions, but good-looking respondents receive significantly higher offers. Using a public goods game in the laboratory, Andreoni and Petrie (2008) showed that attractive people make more money despite contributing the same (on average) as unattractive people. Fletcher (2009) showed that good-looking high school graduates earn 5–10% more than average-looking graduates. More recently, according to a study by Parrett (2015), attractive servers earn more than their less attractive colleagues.

Research has confirmed the contribution of physical appearance to candidate advancement in the recruitment process (López Bóo et al., 2013), earning higher salaries (Andreoni and Petrie, 2008; Parrett, 2015; Aydin and Acun, 2019), and better career prospects (Dion et al., 1972; Shtudiner, 2020). According to Klein and Shtudiner (2020), physical attractiveness has an impact on judging the severity of unethical workplace behavior. In this study, plain-looking employees were evaluated more harshly for “gray area” behavior than attractive employees, confirming the attractiveness-leniency effect. Maestripieri et al. (2017) suggested that attractiveness might lead to overconfidence.

Despite the growing literature, very less is known about the reasons for this beauty premium. Thornhill and Gangestad (1993) and Thornhill and Møller (1997) suggested that physically attractive individuals are healthier. Other studies have found that attractive people are also more intelligent (e.g., Kanazawa, 2011), and intelligence is, in turn, correlated with more pleasing personality traits conducive to interpersonal relations (Chamorro-Premuzic et al., 2006). Moreover, Mobius and Rosenblat (2006) and Rosenblat (2008) concluded that attractive people have better oral skills, better negotiation skills, and greater confidence. The beauty premium documented in the labor market may be linked to these findings.

Mobius and Rosenblat (2006) suggested that good-looking laborers are more confident and, therefore, earn more. Andreoni and Petrie (2008) conducted a laboratory experiment of a repeated linear public goods game and found evidence of stereotyping. Attractive people were perceived/expected to be more cooperative than unattractive people and therefore earned more money. However, when information about the past behavior of each group member was revealed, the beauty premium disappeared, and the participants were less cooperative with them. Using a laboratory trust game, in which trustors viewed the trustees’ photographs, Wilson and Eckel (2006) observed that attractive trustees were perceived as being more trustworthy than unattractive ones, allowing them to earn more money during the first stage of the experiment. Attractive trustees are also expected to return more money than they do in the second stage, so a beauty penalty is incurred when expectations aren’t met. Parrett (2015) found a beauty premium for attractive servers and suggested several explanations: employers’ stereotypes (attractive workers have better attributes such as intelligence); increased confidence that leads to better oral skills and negotiation; and taste-based discrimination.

Some studies involve field experiments to shed more light on the beauty premium. López Bóo et al. (2013) used 2,540 resumes with photographs in response to job postings. They took photographs of fifty real men and women and digitally manipulate them to match with (attractive) or deviate from (unattractive) two golden ratios of facial proportions based on Pallett et al. (2010). They discovered that people who are attractive receive 36% more callbacks than those who are less attractive. This result is robust across six different occupational categories (e.g., finance and sales).

Ruffle and Shtudiner (2015) analyzed the role of attractiveness in the hiring process using more than 5,000 CVs for 2,656 advertised job openings in Israel. They send paired CVs (i.e., one CV with a photograph and one otherwise-identical CV without a photograph) to advertised job postings and found that attractive male candidates tend to be invited to interview significantly more frequently than other male candidates (plain males/no photographs). Surprisingly, the results show that attractive female candidates are significantly less likely to be invited to interview than plain female candidates (18% less) and female candidates without photographs (22.5% less). One possible explanation for the penalization of attractive women (relative to women who do not embed a picture in their CV) is negative signaling. Adding a photograph to a resume is optional rather than required in Israel. Consequently, an attractive woman who attaches a photograph to her CV can be viewed negatively, while an attractive man attaching a photograph to his CV can be perceived as signaling something positive. A follow-up survey and additional analyses suggest that this explanation cannot explain the penalization of attractive women, while substantial evidence indicates female jealousy and envy as part of the explanation (the sample of their respondents was dominated by women). This discrimination against attractive women in the actual market labor is puzzling and deserves more attention.

Ruffle and Shtudiner (2015) were not the first to show that physical attractiveness can also have negative consequences for attractive individuals. Women considered to be good-looking are perceived to be more likely to divorce, have extramarital affairs, and possess certain negative personal traits (Dermer and Thiel, 1975). Hatfield and Sprecher (1986), (Chapter 10) and Agthe et al. (2010, 2011) summarized the downsides for and negative biases against people considered attractive. In an early study by Heilman and Saruwatari (1979), forty-five undergraduates evaluated different job packets that included photographs for potential positions. The authors found that attractive women are discriminated against for certain positions. Lee et al. (2018) conducted four lab experiments to show that it is less likely that attractive candidates will be selected for certain types of jobs. The researchers found that decision-makers believe that attractive candidates would be more dissatisfied with a less desirable job than unattractive candidates. Lin et al. (2018) showed that the presence of attractive servers alters the perception of the taste of the food products consumed. Moreover, an attractive server makes the experience of tasting unpleasant food even more unpleasant.

Shtudiner and Klein’s (2020) research focused on the perception of unethical accountant behaviors and how this judgment is influenced by physical attractiveness. They showed the subjects 18 short vignettes illustrating unethical behavioral dilemmas, each accompanied by a photograph of the individual who committed the act. The subjects were asked to rate the severity of these unethical behaviors. According to the study, subjects were more tolerant of unethical work behaviors conducted by accountants than by other workers. However, they were more judgmental if such behavior violated the law. Also, unethical behavior was tolerated more if the accountant was more attractive.

This article contributes to the existing literature on the beauty premium in the labor market (e.g., see Hamermesh and Biddle, 1994; Mobius and Rosenblat, 2006; Fletcher, 2009; López Bóo et al., 2013; Ruffle and Shtudiner, 2015) in several ways. This article examines a well-known phenomenon in a new professional field. To the best of our knowledge, this is the first study to examine the competitive recruitment process of CPA firms. Hiring the most qualified interns is one of the main profit-maximizing targets of CPA firms, and, therefore, the recruitment process is very important. Our sample also allows us to compare the role of physical appearance between the Big 5 and small- to medium-sized CPA firms. Second, our subject population is unique and includes nearly 300 accountants and human resource managers who make similar decisions in reality, unlike the students surveyed by Heilman and Saruwatari (1979). Third, the recruiting process for accounting positions does not only include an interview with a human resources manager (mostly women) but also include an interview with an accountant, and in some cases a partner in the firm. Therefore, unlike previous studies (Ruffle and Shtudiner, 2015) that included mainly female human resources managers, our sample includes an equal number of men and women and allows for a comparison between the decisions of both genders. The use of a controlled environment allows us to control for other managers’ characteristics such as gender, seniority, and age. Fourth, our design permits us to eliminate the signaling effect associated with attaching a photograph from the analysis of the effect of physical appearance (Ruffle and Shtudiner, 2015). Fifth, we also presented evidence that physical attractiveness is positively related to the desirable traits of successful accountants. This finding is in accordance with the vast body of research in psychology that shows that people attribute attractive men and women with a wide range of positive characteristics and dispositions (Feingold, 1992).

## Design

### Ratings of candidates’ traits

An analysis of facial attractiveness in accounting internship candidates is presented in this study, and using the website http://www.faceresearch.org, we selected 42 headshots of men and women. Each of the headshots was of a young, adult Caucasian student. We presented the photographs to 809 accounting students (402 men, 407 women; mean age = 25.9 years, s.d. = 3.4) and requested them to rate the photographs for the six essential traits for accountants and attractiveness on a 1–9 scale [we used a 1–9 scale as in Andreoni and Petrie (2008), Ruffle and Shtudiner (2015), and Ruffle et al. (2022)]. No identifying information was displayed with the photographs. The subjects were requested to rate five images of men and five images of women, and each photograph appeared in more than one questionnaire version.

While we are primarily interested in attractiveness, we also asked the students to assess the ethnicity of the individuals. Due to evidence showing discrimination against Jewish immigrants of North African and Middle Eastern origin (i.e., Sephardic Jews) compared with Europeans and Ashkenazis, ethnicity rating plays an important role (see Malul et al., 2010; Rubinstein and Brenner, 2014). To avoid any bias, versions of questionnaires were prepared that differed in terms of the order of photographs, the order of photographed person’s gender, and the order of the characteristics evaluated. Each image appeared in at least two versions of the questionnaire, each time in a different position in the series.

Table 1 describes the mean, standard deviation, and median ratings of each photograph’s attractiveness on a 1–9 scale, where 9 represents most attractive. The mean rating for men (4.84) was lower than that for women (5.28). The difference between these two groups was significant based on a t-test (T = −7.8, p = 0.000, two-tailed t-test). This finding that women tend to be rated higher than men in terms of beauty is in line with the existing literature (see Hamermesh, 2011).

**TABLE 1:** Summary statistics of attractiveness ratings.

Men’s photographs				Women’s photographs			
Headshot	Mean	Standard deviation	Median	Headshot	Mean	Standard deviation	Median
1	6.02	2.41	7	22	6.80	2.27	8
2	3.97	2.43	4	23	5.74	2.23	6
3	4.98	2.21	5	24	3.82	2.00	4
4	4.79	2.35	5	25	4.95	2.23	5
5	3.75	2.43	3	26	6.22	2.39	7
6	4.65	2.21	5	27	4.82	2.41	4
7	4.43	2.20	4	28	4.17	1.98	4
8	4.07	2.53	4	29	7.40	1.99	8
9	4.61	2.15	5	30	4.91	2.19	5
10	5.05	2.31	5	31	5.65	2.21	6
11	5.83	2.52	6	32	4.85	2.47	5
12	4.20	2.28	4	33	5.30	2.27	6
13	4.70	2.47	5	34	5.22	2.50	5
14	5.55	2.59	6	35	4.78	2.42	5
15	5.04	2.49	5	36	3.51	2.40	3
16	4.43	2.19	4	37	3.90	2.08	4
17	4.98	1.80	5	38	4.84	1.84	4
18	6.79	2.03	8	39	4.37	2.10	4
19	3.24	1.98	3	40	6.60	2.13	7
20	7.08	1.81	8	41	4.68	2.13	4.5
21	4.12	2.51	3	42	6.21	2.05	6
Overall	4.84	2.47	5	Overall	5.28	2.47	5

This table displays the summary statistics of attractiveness ratings provided by 809 accounting students. On a 1–9 scale (9 attractive, 1 unattractive), 42 headshots were ranked. Candidates are identified by their photograph number for internal use.

### Ratings of tendency to employ

Our aim was to determine if physical attractiveness plays a role in the hiring process by displaying the photographs to 298 accountants and human resources professionals working in Israeli CPA firms. According to their instructions, the photographs belonged to candidates who applied for accounting internship positions at their firms, and each of them met the requirements of the job. Using a Likert scale, the managers rated the likelihood of hiring each candidate from 1 (unlikely to hire) to 9 (likely to hire). Participants assessed 15 photographs of women and 15 photographs of men. To avoid any bias, versions of questionnaires were prepared that differed in terms of the order of photographs and the order of photographed person’s gender.

Table 2 displays the distribution of the managers and the firms in which candidates are to be hired. According to the table, a similar number of female (52%) and male (48%) managers responded to the questionnaire. Almost half of the subjects were in a senior position (48%); most were accountants (83%), while the rest (17%) were human resources professionals. The distribution according to the size of firms reveals that 49% of the managers worked at Big 5 firms and the remaining 51% worked at small- to medium-sized firms.

**TABLE 2:** Distribution of managers and firms.

Variable	Category	Num. of observations	Proportion
Gender	Men	144	0.48
	Women	154	0.52
Seniority	Senior	142	0.48
	Junior	108	0.36
	Unspecified	48	0.16
Occupation	Accountancy	247	0.83
	Human Resources	51	0.17
Size of Firm	Big 5	146	0.49
	Small-Mid size	152	0.51

This table presents the distribution of 298 managers (accountancy or HR) in four categories, namely, gender, seniority, occupation, and size of firm.

## Results

### Main results: The impact of attractiveness on the likelihood to be hired

**FIGURE 1:**
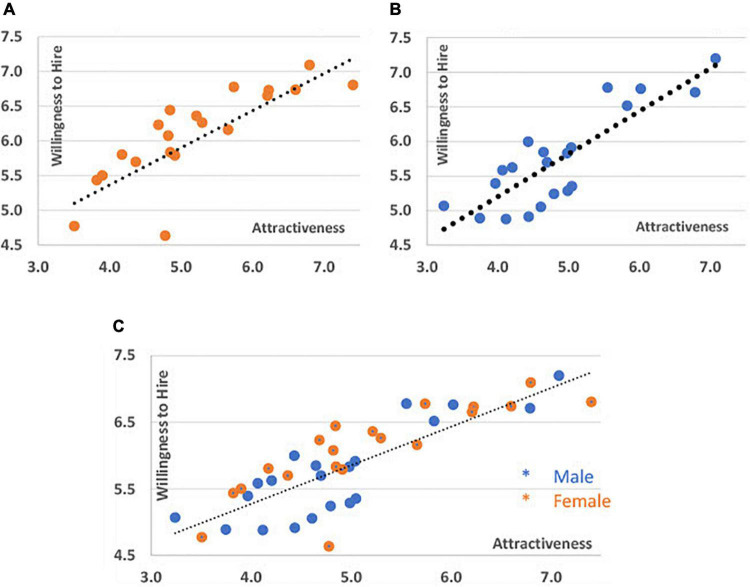
Willingness to hire and attractiveness. This figure shows the willingness to hire (Y) and attractiveness (X) by male candidates [panel **(A)** ], female candidates [panel **(B)** ], and all candidates [panel **(C)** ]. Each point represents the average score of a single candidate rated by 298 employers (willingness to hire) and 809 students (attractiveness).

We began by asking: Does a candidate’s attractiveness affect his or her chances of being hired? Each candidate’s average attractiveness and average employment likelihood are displayed in Figure 1. Each of them is represented by a point (overall 42), with divisions between female candidates (Panel A), male candidates (Panel B), and all candidates (Panel C). The likelihood of being employed is positively correlated with attractiveness on all trend lines.

We estimated the following equation in order to control additional explanatory variables:


(1) $$WouldYouHire_{ij}=\propto_{0}+\beta_{1}\text{⋅}Attractive_{i}+\beta_{2}\text{⋅}Candidate$$

$$\;Female_{i}+\beta_{3}\text{⋅}CandidateEthnicity_{i}$$

$$\;+\beta_{4}\text{⋅}ManagerFemale_{j}+\beta_{5}\text{⋅}$$

$$\;Age_{j}+\beta_{6}\text{⋅}HumanResources_{j}+\beta_{7}\text{⋅}Senior_{j}$$

$$\;+\beta_{8}\text{⋅}Big5_{j}+\eta_{j}+\varepsilon_{ij}$$

Manager j’s interest in hiring candidate i is represented by the dependent variable WouldYouHire_ij_, on a scale from 1 to 9. Attractive_i_ is the key independent variable and represents the average rating of candidate i, on a scale of 1 (unattractive) to 9 (attractive). The dummy variable Candidate Female_i_ (Manager Female_j_) gets a value of 1 if candidate i (manager j) is female; otherwise, it gets a value of 0. Candidate i’s average rating of ethnic origin is represented by the variable Candidate Ethnicity_i_, on a scale of 1 to 9 (1 for “definitely Sephardi,” 9 for “definitely Ashkenazi,” and 5 for “uncertain”). Age_j_ is the manager’s age, and the dummy variable Human Resources_j_ is assigned a value of 1 if the occupation of the manager j is in human resources, and 0 if the occupation is in accountancy. The dummy variable Senior_j_ is assigned a value of 1 if manager j is senior; otherwise, it is assigned a value of 0. The dummy variable Big5_j_ is assigned a value of 1 if manager j works at one of the five largest firms; otherwise, it is assigned a value of 0. The unobserved heterogeneity η_j_ represents the managers’ characteristics unobservable in the data but reflected in the dependent variable, WouldYouHire_ij_. The stochastic error term is ε_ij_. We also estimated a short variation of Eq. (1) (without controls, see Table 3).

**TABLE 3:** The effect of attractiveness on the tendency to hire a candidate.

	(1)	(2)	(3)	(4)	(5)	(6)
Attractive	0.606***	0.591***	0.639***	0.554***	0.537***	0.760***
	(0.037)	(0.041)	(0.048)	(0.058)	(0.061)	(0.076)
Candidate female		0.047	0.513**	0.517**	0.231	0.853**
		(0.056)	(0.238)	(0.238)	(0.320)	(0.356)
Candidate ethnicity		0.062***	0.056***	0.056***	0.064***	0.046***
		(0.013)	(0.012)	(0.012)	(0.017)	(0.017)
Manager female		0.111	0.111	−0.825*		
		(0.142)	(0.142)	(0.474)		
Age		0.021**	0.021*	0.021*	0.042***	−0.006
		(0.011)	(0.011)	(0.011)	(0.014)	(0.013)
Human resources		0.398**	0.397**	0.396**	0.569**	0.237
		(0.167)	(0.167)	(0.167)	(0.232)	(0.241)
Senior		0.069	0.069	0.069	0.159	−0.062
		(0.153)	(0.153)	(0.153)	(0.205)	(0.224)
Big 5		−0.068	−0.069	−0.068	−0.012	−0.175
		(0.147)	(0.147)	(0.148)	(0.189)	(0.219)
Att. * Candidate female			−0.092**	−0.093**	−0.047	−0.146**
			(0.044)	(0.044)	(0.060)	(0.066)
Att. * Manager female				0.185**		
				(0.081)		
Observations	8,939	7,470	7,470	7,470	3,990	3,480
R-squared	0.089	0.096	0.096	0.098	0.095	0.112
Manager	All	All	All	All	Male	Female
Candidate	All	All	All	All	All	All
Controls	N	Y	Y	Y	Y	Y

This table displays the results of the panel data regression: *WouldYouHire_ij_* = ∝_0_ + β_1_⋅*Attractive_i_* + β_2_⋅*Candidate Female_i_* + β_3_⋅*Candidate Ethnicity_i_* + β_4_⋅*Manager Female_j_* + β_5_⋅*Age* + β_6_⋅*Human Resources_j_* + β_7_⋅*Senior_j_* + β_8_⋅*Big*5*_j_* + η*_j_* + ε*_ij_*. Robust standard errors are presented in parentheses. *, **, and *** denote significance at the 10%, 5%, and 1% levels, respectively.

Since each photograph is rated by several managers, and managers are observed at various points and rank several candidates, the methods for estimating panel data models (Greene, 2012) are appropriate. It is useful to use panel data models since we suspect that the outcome variable may depend on explanatory variables, which are not observed but are correlated with the observed explanatory variables. In other words, the observations are independent across groups (clusters), but not necessarily within groups. This estimation method does not affect the estimated coefficients (but affects the variance-covariance matrix of the estimators and standard errors). The equation was estimated by a random effects model, in which the individual-specific effect is a random variable that is uncorrelated with the explanatory variables.^2^

Table 3 reports the impact of a candidate’s attractiveness on the likelihood to be hired, as estimated from Eq. (1). Columns 1–4 display the results for managers and candidates of both genders. These regressions are different in that they include hierarchically different explanatory variables. Column 1 in Table 3 shows that the more attractive the candidate, the higher the tendency to hire him/her. Adding our set of controls in Column 2 (candidate’s gender, candidate’s ethnicity, manager’s gender, manager’s age, occupation, seniority, and dummy variable that gets 1 for Big 5 firms) does not change the result. The variable Attractive_i_ is positive and significant (p = 0.000) both with (0.59) and without (0.61) controls, suggesting the existence of a beauty premium. The positive and significant coefficient of Candidate Ethnicity indicates discrimination against Sephardic candidates. Adding the interaction variable Attractive . Candidate Female_i_ (Column 3) reveals that the attractiveness premium for females is significantly lower compared to males (−0.09).

An interesting question arises as to whether female and male managers assign a different weight for attractiveness in the recruitment process. Adding the interaction variable Attractive Manager Female_i_ (Column 4) reveals that the beauty premium is significantly greater among female managers (0.185), implying that good-looking candidates will have better chances to get hired if the interview is done by a female manager.

The evidence provided by Ruffle and Shtudiner (2015) supports the idea that female jealousy and envy may be contributing factors to female managers being less affected by the beauty of female candidates than by the beauty of male candidates. To test this, we also restricted the sample to male (Column 5) and female (Column 6) managers. In Column 5 (male managers), we found that female and male candidates receive the same beauty premium, and the interaction coefficient (Attractive . Candidate Female_i_) is not significant. However, when female managers do the hiring (Column 6), the beauty premium for female candidates relative to male candidates is significantly lower, and the interaction coefficient (Attractive . Candidate Female_i_) is negative (−0.146) and significant. These results are in line with Ruffle and Shtudiner (2015). Alongside the finding that Candidate Female’s coefficient is significant and positive, it appears that women discriminate in favor of other women, but in comparison with attractive men, attractive women receive a lower beauty premium.

The regressions included two perceived characteristics as independent variables, namely, Attractive and Candidate Ethnicity. To create a single beauty measure and a single ethnicity measure, over all photographs rated by the same person, we calculated the mean rating and standard deviation for each trait j. Next, we subtracted rater n’s specific rating of candidate i’s photograph from the above mean trait j rating and divided it by the standard deviation. We then computed the mean of all N ratings for this candidate i and trait j. That is, the mean standardized rating for candidate i’s trait j is given by the expression:


$$\frac{\sum_{n=1}^{N}\left[\left(x_{ijn}-\sum_{i=1}^{T}x_{ijn}\right)/\sqrt{{\left(x_{ijn}-\sum_{i=1}^{T}x_{ijn}\right)}^{2}}\right]}{N}.$$

Since some raters can give consistently higher ratings compared with other raters, we also reported (in Supplementary Material) the standardized ratings. The results are robust to replacing the variables of attractiveness and ethnicity with standardized ones. Table 3 with the standardized variables is presented in Supplementary Table 1.

### Likelihood to be hired and dichotomous attractiveness

**FIGURE 2:**
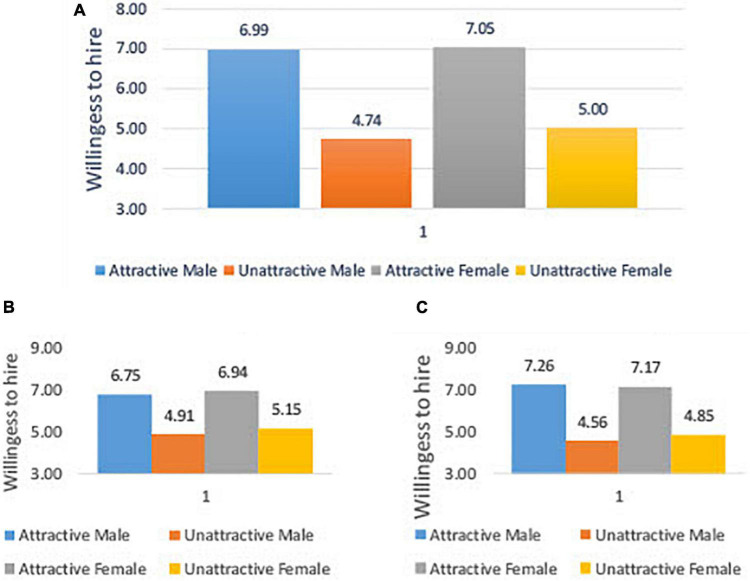
Willingness to hire an attractive/unattractive candidate. This figure shows the willingness to hire (Y) attractive/unattractive male/female candidates. Panel **(A)** shows the willingness to hire of both male and female managers while panel **(B)** [panel **(C)**] shows the willingness to hire of only male (female) managers. Based on the average score of attractive (unattractive) female candidates rated by 502 (502) managers and the average score of attractive (unattractive) male candidates rated by 456 (400) managers.

More studies that deal with attractiveness and probability of being hired use a dichotomous variable for beauty, very attractive versus very unattractive [e.g., Ruffle and Shtudiner (2015)]. Therefore, we also provided a dichotomous analysis of beauty. We used a subsample of the two most attractive and two most unattractive candidates for each gender. Figure 2 describes the average score of the willingness to hire attractive/unattractive male candidates (6.99/4.74) and attractive/unattractive female candidates (7.05/5.00). Panel B and Panel C describe the willingness to hire male and female managers, respectively. These results support our findings that female managers value attractiveness more than male managers do.

To strengthen this finding, we also created a dichotomous attractiveness variable (*D_Attractive*) that gets 1 for the two most attractive candidates and 0 for the two most unattractive candidates. The results of the regression (Eq. 1) with *D_Attractive* are summarized in Table 4. Using the same control variables as in Table 3, we found the same results: good-looking candidates have a better chance of being hired; attractive female candidates have a higher chance of being accepted than attractive male candidates (Column 2: 0.551*); and female managers tend to put more weight on attractiveness (Column 3: 0.724^**^). In contrast to our previous results) Table 3, regressions 5–6), we found that male managers discriminate in favor of (very) attractive female candidates over (very) attractive male candidates (Column 5: 0.820^**^) while female managers do not (Column 7: 0.200).

**TABLE 4:** The effect of dichotomous attractiveness on the tendency to hire a candidate.

	All managers			Male managers		Female managers	
	(1)	(2)	(3)	(4)	(5)	(6)	(7)
D_Attractive	2.184***	2.041***	1.853***	1.849***	1.639***	2.587***	2.535***
	(0.159)	(0.177)	(0.206)	(0.216)	(0.239)	(0.231)	(0.256)
D_Attractive * Candidate female		0.551*			0.820**		0.200
		(0.285)			(0.391)		(0.417)
D_Attractive * Manager female			0.724**				
			(0.284)				
Controls	Y	Y	Y	Y	Y	Y	Y
Observations	1,860	1,860	1,860	1,004	1,004	856	856
R-squared	0.1844	0.1854	0.1898	0.1476	0.1500	0.2398	0.2400

This table presents the effect of *dichotomy attractiveness* (*D_Attractive*) on the tendency to hire a candidate. *D_Attractive* is a dummy variable that gets 1 for a very attractive candidate and 0 for a very unattractive candidate. The control variables are candidate gender, candidate ethnicity, manager age, manager gender, human resources, firm size, and seniority. We use random effects and clusters. Robust standard errors are presented in parentheses. Note that *, **, and *** denote significance at the 10%, 5%, and 1% levels, respectively.

### The role of firm size

Next, we investigated the effect of firm size on the beauty premium by estimating the model in Eq. (1) for the Big 5 firms and the other firms separately. Columns 1 and 2 in Table 5 show that there is a statistically significant beauty premium (1%) in Big 5 firms (0.69) and small- to medium-sized firms (0.51). The interaction term between *Attractive* and *Big5* is included in Column 3. The coefficient of this variable is positive (0.19) and significant, indicating that the beauty premium has an enhanced impact on Big 5 firms. Supplementary Table 2 shows the results using standardized attractiveness and ethnicity.

**TABLE 5:** The effect of attractiveness on the tendency to hire a candidate in Big 5 vs. other firms.

	Big 5	Small- to medium-sized firms	All firms
	(1)	(2)	(3)
Attractive	0.687***	0.510***	0.537***
	(0.058)	(0.057)	(0.054)
Attractive * Big 5			0.189**
			(0.074)
Observations	3,571	3,899	8,880
Controls	Y	Y	Y
Overall R-squared	0.116	0.081	0.106

The dependent variable is represented by the tendency to hire (1–9 points Likert scale). Robust standard errors are presented in parentheses, and **, and *** denote significance at the 5%, and 1% levels, respectively.

### The role of managers’ seniority and occupation

We also investigated the effect of managers’ seniority (Table 6, Columns 1−3) and occupation (Table 6, Columns 4−6) on the beauty premium by estimating the model in Eq. (1). Columns 1–3 show that the beauty premium is statistically significant (1%) among both senior (0.55) and junior (0.63) managers. An interaction term between *Attractive* and *Senior* is included in Column 3, and its coefficient (−0.10) is not significant, implying that senior managers make decisions based on attractiveness just as junior managers do. Further research is needed to answer the question of whether it implies that senior managers had positive experiences when they hired more attractive employees in the past, and therefore, they continue hiring attractive employees. The alternative is that this bias is simply difficult to overcome, no matter the experience one has had with previous hiring decisions.

**TABLE 6:** The effect of attractiveness on the tendency to hire a candidate by managers’ seniority and occupation.

	Seniority			Occupation		
	Senior	Junior	All managers	Accountants	Human resources	All managers
	(1)	(2)	(3)	(4)	(5)	(6)
Attractive	0.545***	0.630***	0.636***	0.593***	0.591***	0.593***
	(0.059)	(0.057)	(0.056)	(0.090)	(0.046)	(0.046)
Attractive * Senior			−0.098			
			(0.081)			
Attractive * Occupation						−0.005 (0.101)
Observations	3,209	4,261	7,470	6,000	1,470	7,470
Controls	Y	Y	Y	Y	Y	Y
Overall R-squared	0.082	0.112	0.097	0.111	0.091	0.096

The dependent variable is represented by the tendency to hire (1–9 points Likert scale). Robust standard errors are presented in parentheses, and *** denotes significance at the and 1% level.

Columns 4–6 show that the beauty premium is statistically significant (1%) among both human resources (0.59) and accounting (0.59) managers. An interaction term between *Attractive* and *Human Resources* is included in Column 6, and its coefficient (−0.01) is not significant, implying the beauty premium is similar for both occupations. Supplementary Table 3 shows the results using standardized attractiveness and ethnicity.

### Possible explanation for the beauty premium

Finally, we investigated a possible explanation for the existence of a beauty premium. There is a large body of psychology literature that shows that people attribute attractive men and women a wide array of positive traits and dispositions (see Feingold, 1992). Therefore, in our exploratory data analysis, the correlation between six essential characteristics of accountants and attractiveness was calculated (Table 7). The correlations are all significantly higher than zero, and it is robust for both groups of subjects (students and managers) and candidates’ gender (Rows 2−5). We also regressed the attractiveness rating on each trait separately. All the coefficients are positive and significant (1% level).

**TABLE 7:** Correlations between attractiveness and other perceived characteristics.

Groups	Ethical	Analytical	Detail oriented	Inquisitive	Intuitive	Critical	Obs.
All data	0.242***	0.247***	0.270***	0.257***	0.307***	0.313***	7542
Male-rank-male	0.171***	0.194***	0.208***	0.236***	0.277***	0.241***	1805
Male-rank-female	0.228***	0.299***	0.284***	0.238***	0.300***	0.342***	1946
Female-rank-male	0.276***	0.211***	0.262***	0.247***	0.329***	0.318***	1818
Female-rank-female	0.285***	0.315***	0.333***	0.317***	0.333***	0.350***	1973

***p < 0.01.

## Conclusion and discussion

Our study shows that CPA firms in Israel are more likely to accept attractive candidates for internship programs. This result was found both in continuous and dichotomous analyses of beauty, and it shows that in addition to wage discrimination and promotion decisions, beauty discrimination also occurs at the earliest stages of hiring. Even though previous research has shown there to be a beauty premium, it is somewhat surprising that, even among accounting firms where professionalism is well defined, a factor that has no functional role such as physical appearance has such a strong influence on hiring decisions.

This bias is the result of the halo effect, whereby judgments concerning unknown traits are influenced by overall impression (Nisbett and Wilson, 1977). Despite the prevailing perception that women are more likely to be judged based on their appearance, we observed that women receive a lower beauty premium than men.

Due to the sample and survey design, we were able to compare the decisions of female and male managers and observe whether these decisions were influenced by attractiveness differently. The beauty premium is significantly greater among female managers,^3^ and the beauty premium for female candidates relative to male candidates is significantly lower in that case. This result is in line with Ruffle and Shtudiner (2015), who suggested that female jealousy and envy are possible explanations for discriminatory attitudes from women toward other women. However, in opposition to their research, we did not find a beauty penalty for attractive women. Possible explanations for this difference are the type of experiment conducted (field versus laboratory) and the signaling of attaching a photograph to a resume, which is included in their research but not in ours. In certain work cultures where it is optional to attach a photograph to one’s CV, the decision to do so may be viewed differently depending on an individual’s attractiveness and gender.

Our design allows to overcome one of the main weaknesses of Callback experiments. In Callback experiments, the researchers cannot identify the interviewer gender. They can only identify the voice of the person who makes the call. This may be the decision-maker or just a secretary. Using our sample and design, we could compare the decisions of female and male managers with much better accuracy. This advantage doesn’t come for free. One may claim that providing only photographs to the interviewer may lead the interviewer to rank candidates by beauty (as this is the only available information) which leads to a beauty premium. But even if this is true, it cannot explain the different outcomes between male and female managers, and we can still learn about the gap in beauty premium between female and male managers, and therefore, our contribution goes behind this obstacle.

As we found by comparing the Big 5 versus smaller firms, the beauty premium exists in both groups, but it is stronger in the former. One possible explanation for this difference is that big-company positions are more competitive and managers may use signals such as a candidate’s physical appearance and the associations it evokes to determine whether the candidate is qualified for the job. The surprising level of beauty-based discrimination in small firms suggests that the phenomenon likely extends to other employment markets.

We suggest a possible explanation for this beauty premium: an attractive person is believed to possess characteristics that make him or her a successful accountant and, therefore, have a higher probability of being hired. In light of these results, there is an important implication: ineffective elimination of suitable intern candidates can result from discrimination based on attractiveness in the hiring process.

One way to reduce the effect of beauty is by providing counter-stereotype training. This is hard to achieve in a small firm but might be of a value to big firms where we found a higher beauty premium. Counter-stereotype training can raise awareness of beauty-based discrimination and may help reduce not only beauty premium but also different types of stereotypes. For example, Burns et al. (2017) showed that when participants are aware of their own bias, they succeed in reducing it significantly. On a different level, removing photographs from CV will prevent beauty-based discrimination (at least in the callback stage).

## Data availability statement

The datasets are available on request. The raw data supporting the conclusions of this article will be made available by the authors without undue reservation.

## Ethics statement

Ethical review and approval was not required for the study on human participants in accordance with the local legislation and institutional requirements. The patients/participants provided their written informed consent to participate in this study.

## Author contributions

OS and ZS designed the experiments, prepared the materials, performed the experiments, analyzed the data, and wrote the manuscript. Both authors contributed to the article and approved the submitted version.

## Footnotes

1 Globes, 9.5.21, https://www.globes.co.il/news/article.aspx?did=1001370226 (Hebrew).

2 We chose a random effects model and not fixed effects model since the purpose of the study is to compare the candidates, and the intent is to generalize to other managers. Thus, manager should be considered as a random factor, not a fixed factor.

3 According to the existing literature, it is not completely clear whether physical attractiveness is more important to men or women. The meta-analysis by Feingold (1990), for example, shows that men value physical attractiveness more than women, particularly when romantic attraction is involved. However, other studies show that females are more beauty-conscious than males (e.g., Kahn et al., 1971; Parrett, 2015).
