# Supplementary material for: Beauty is in the eye of the employer: Labor market discrimination of accountants
Source: Front Psychol. 2022 Jul 29;13:928451. doi: 10.3389/fpsyg.2022.928451 (PMC9372560; doi:10.3389/fpsyg.2022.928451)
Supplement: Supplementary file 1 [file Data_Sheet_1.docx]

Appendix

**Table A1: The Effect of Attractiveness on the Tendency to Hire a Candidate using standardized ratings**

|  | (1) | (2) | (3) | (4) | (5) | (6) |
| --- | --- | --- | --- | --- | --- | --- |
| Attractive | 1.333*** | 1.279*** | 1.434*** | 1.250*** | 1.216*** | 1.694*** |
|  | (0.082) | (0.089) | (0.106) | (0.125) | (0.131) | (0.168) |
| Candidate Female |  | 0.068 | 0.065 | 0.065 | 0.004 | 0.135* |
|  |  | (0.056) | (0.056) | (0.056) | (0.078) | (0.079) |
| Candidate Ethnicity |  | 0.144*** | 0.111*** | 0.112*** | 0.156*** | 0.061 |
|  |  | (0.038) | (0.036) | (0.036) | (0.051) | (0.052) |
| Manager Female |  | 0.109 | 0.109 | 0.118 |  |  |
|  |  | (0.142) | (0.142) | (0.141) |  |  |
| Age |  | 0.021** | 0.021** | 0.021** | 0.042*** | -0.006 |
|  |  | (0.011) | (0.011) | (0.011) | (0.014) | (0.013) |
| Human Resources |  | 0.410** | 0.410** | 0.410** | 0.580** | 0.253 |
|  |  | (0.167) | (0.167) | (0.167) | (0.232) | (0.241) |
| Senior |  | 0.079 | 0.079 | 0.078 | 0.169 | -0.051 |
|  |  | (0.153) | (0.153) | (0.153) | (0.205) | (0.224) |
| Big 5 |  | -0.072 | -0.072 | -0.072 | -0.015 | -0.179 |
|  |  | (0.147) | (0.148) | (0.148) | (0.189) | (0.220) |
| Att. * Candidate Female |  |  | -0.301*** | -0.303*** | -0.202 | -0.423*** |
|  |  |  | (0.095) | (0.095) | (0.127) | (0.142) |
| Att. * Manager Female |  |  |  | 0.405** |  |  |
|  |  |  |  | (0.178) |  |  |
| Observations | 8,939 | 7,470 | 7,470 | 7,470 | 3,990 | 3,480 |
| R-squared | 0.089 | 0.096 | 0.097 | 0.100 | 0.096 | 0.114 |
| Manager | All | All | All | All | Male | Female |
| Candidate | All | All | All | All | All | All |
| Controls | N | Y | Y | Y | Y | Y |

Note: Table 1 displays the results of the panel data regression: ${WouldYouHire}_{ij}= \propto_{0}+\beta_{1}\cdot{Attractive standardized}_{i}+\beta_{2}\cdot{Candidate Female}_{i} +\beta_{3}\cdot{Candidate Ethnicity standardized}_{i}+\beta_{4}\cdot{Manager Female}_{j} +\beta_{5}\cdot Age+\beta_{6}\cdot{Human Resources}_{j}+\beta_{7}\cdot{Senior}_{j}+\beta_{8}\cdot{Big5}_{j}+\eta_{j}+\varepsilon_{ij}$. Robust standard errors are presented in parentheses. *, **, *** denote significance at the 10%, 5%, and 1% levels respectively.

**Table A2: The Effect of Attractiveness on the Tendency to Hire a Candidate
in Big 5 vs Other Firms using standardized ratings**

|  | Big 5 | Small- to Medium-sized  Firms | All Firms |
| --- | --- | --- | --- |
|  | (1) | (2) | (3) |
| Attractive | 1.470*** | 1.115*** | 1.169*** |
|  | (0.125) | (0.125) | (0.120) |
| Attractive * Big 5 |  |  | 0.388** |
|  |  |  | (0.163) |
| Observations | 3,571 | 3,899 | 8,880 |
| Controls | Y | Y | Y |
| Overall R-squared | 0.115 | 0.083 | 0.106 |

Notes: The dependent variable is represented by the tendency to hire (1–9 Likert scale). Robust standard errors are presented in parentheses, and *, **, and *** denote significance at the 10%, 5%, and 1% levels, respectively.

**Table A3: The Effect of Attractiveness on the Tendency to Hire a Candidate
by Managers' Seniority and Occupation using standardized ratings**

|  | Seniority | | | Occupation | | |
| --- | --- | --- | --- | --- | --- | --- |
|  | Senior | Junior | All Managers | Accountants | Human Resources | All Managers |
|  | (1) | (2) | (3) | (4) | (5) | (6) |
| Attractive | 1.193*** | 1.349*** | 1.361*** | 1.288*** | 1.276*** | 1.277*** |
|  | (0.128) | (0.123) | (0.122) | (0.191) | (0.101) | (0.100) |
| Attractive * Senior |  |  | -0.183 |  |  |  |
|  |  |  | (0.179) |  |  |  |
| Attractive * Occupation | |  |  |  |  | \| 0.002 \| \| --- \| \| (0.101) \| |
| Observations | 3,209 | 4,261 | 7,470 | 6,000 | 1,470 | 7,470 |
| Controls | Y | Y | Y | Y | Y | Y |
| Overall R-squared | 0.084 | 0.111 | 0.097 | 0.112 | 0.091 | 0.096 |

Notes: The dependent variable is represented by the tendency to hire (1–9 Likert scale). Robust standard errors are presented in parentheses, and *, **, and *** denote significance at the 10%, 5%, and 1% levels, respectively.
